# Chemical Phenomena of Digestion

**Published:** 1842-07-16

**Authors:** 


### ANALECTA.

*Chemical Phenomena of Digestion.*—From various experiments made by MM. Sandras and Bouchardat, they conclude,—

1. That the digestive function of the stomach consists (as far as albuminous substances are concerned) in dissolving these substances in hydrochloric acid.

2. This acid, diluted to the 500th degree, is sufficient to dissolve the above mentioned substances, provided they are not in a crude state; when they are boiled, diluted hydrochloric acid is unable to dissolve them in glass vessels; hence, since we find them dissolved in the stomach, something more than a mere process of solution must take place in the stomach; still the presence of hydrochloric acid appears to be indispensable.

3. The digestion and absorption of albuminous matters take place almost exclusively in the stomach.

4. The solution of fecula also seems to be confined to the stomach; in the ordinary state it does not appear to be changed into sugar; nor is it proved that it is transformed into starch; the authors, however, regard its transformation into lactic acid as demonstrated.

5. The absorption of fecula does not appear to be confined to the stomach, as that of albuminous matter is; and this accords with the peculiar arrangement of the stomach in non-carnivorous animals.

6. Fatty substances are not attacked by the stomach; they pass on into the duodenum, where they form a kind of emulsion with the alkalis furnished by the liver and pancreas; this emulsion exists in abundance throughout the intestinal canal.

7. The chyle appeared to be somewhat less abundant in quantity, but identical in properties, when taken from fasting animals, and from those fed on albuminous substances, or fecula. When the animal, however, was fed on fat, it presented a marked difference, the fat being found in considerable proportion in chyle.

The deductions which the authors draw from the foregoing facts are as follows:—It is generally admitted that alimentary substances are transformed by the stomach into a particular matter, called chyme. The authors think that this chyme is a mixture of alimentary residua which have not been dissolved, with secretions from the intestinal canal, and that it is not a fluid prepared for assimilation. The chyle was regarded as being composed of alimentary matter very minutely divided, or dissolved afresh. But colored fibrin does not give colored chyle, and the chyle collected during the digestion of starch is the same as that collected during the digestion of fibrin.

Hence it appears probable that albuminous and feculent substances are not transformed into chyle. The experiments of the authors, also, would lead to the conclusion that the orifices of the chyliferous vessels are intended to absorb the fatty substances turned into emulsions by the bile. But the chyle has another office. When palatable substances are presented to an animal, the gastric juice poured out into the stomach contains a remarkable proportion of hydrochloric and lactic acids, evidently furnished by the decomposition of chloride of sodium and lactate of soda; during this time the abdominal glands prepare chyle, the alkaline nature of which predominates in proportion to the acidity of the gastric juice, and the chyle mixes with the blood, so as to neutralise exactly the acid necessary for the solution of alimentary substances.—*Trans. Acad. of Sci. of Paris*, from *Provin. Med. Jour.*, May 21, 1842.

